# Routine MRI for opportunistic osteoporosis screening: diagnostic accuracy of vertebral bone quality and a novel femoral bone quality score compared with DXA

**DOI:** 10.1007/s00256-026-05330-z

**Published:** 2026-08-08

**Authors:** Margrit Elis Müller, João Paulo Colhado Ferreira, Laura Mulazzani Minuzzi Macedo, Lucia Li, Akemi Osawa, Leonardo Kazunori Tsuji, Daisy Terumi Kase, Lucas Kenzo Miyahara, André Yui Aihara, Adham do Amaral e Castro

**Affiliations:** 1 Present Address: Department of Diagnostic Imaging, Escola Paulista de Medicina, Federal University of São Paulo (UNIFESP), Graduate Program Clinical Radiology, Federal University of São Paulo (UNIFESP), São Paulo, Brazil; 2 Department of Radiology and Diagnostic Imaging, Hospital Israelita Albert Einstein, São Paulo, Brazil; 3 DASA Teaching and Research Institute, Diagnóstico das Américas (DASA), São Paulo, SP Brazil

**Keywords:** Osteoporosis, Osteopenia, Vertebral bone quality, DXA, MRI

## Abstract

**Objectives:**

To evaluate vertebral bone quality (VBQ) and femoral bone quality (FBQ) scores as potential imaging markers of bone mineral density (BMD) for opportunistic osteoporosis screening in at‑risk patients and to assess their correlation with DXA *T*-scores.

**Methods:**

This retrospective cross-sectional study included women ≥ 40 years and men ≥ 50 years who underwent DXA and lumbar spine, hip, or pelvic MRI within 60 days. All MRI examinations were performed at 1.5 T. VBQ and FBQ were obtained using manually placed circular ROIs. FBQ was derived from coronal T1-weighted images using two approaches: femoroacetabular joint fluid (FBQfa) and cerebrospinal fluid (FBQcsf). Interobserver reproducibility was assessed using intraclass correlation coefficient (ICC). Correlations with DXA *T*-scores and diagnostic performance for osteoporosis were analyzed using Pearson coefficients and ROC curves.

**Results:**

Among 248 patients, VBQ demonstrated excellent reproducibility (ICC = 0.922) and a weak-to-moderate negative correlation with DXA *T*-scores (*r* = −0.307; *p *< 0.001). VBQ values increased across DXA bone density categories, with an optimal cutoff of 3.61 for osteoporosis (AUC = 0.71). FBQcsf showed high reproducibility (ICC = 0.874) compared with FBQfa (ICC = 0.462) but no evidence of correlation with DXA (*p* = 0.151). FBQcsf distinguished normal BMD from osteopenia (*p* = 0.002) and, after adjustment for age and sex, also differentiated patients with osteopenia from those with osteoporosis (*p* = 0.011).

**Conclusion:**

VBQ can serve as an opportunistic exploratory screening marker of low BMD. FBQcsf may complement VBQ by identifying early marrow-related changes, although further validation is required.

**Supplementary information:**

The online version contains supplementary material available at https://doi.org/10.1007/s00256-026-05330-z.

## Introduction

Osteoporosis is a common systemic skeletal disease characterized by reduced bone mineral density (BMD) and deterioration of bone microarchitecture, leading to increased bone fragility and fracture risk. It predominantly affects postmenopausal women and is a major contributor to morbidity in the aging population [1].

Dual-energy X-ray absorptiometry (DXA) is the current gold standard for assessing BMD and diagnosing osteoporosis. Despite established screening recommendations, DXA remains underutilized, and osteoporosis continues to be underdiagnosed [1, 2]. DXA provides a two-dimensional projectional measurement and cannot evaluate bone microarchitecture, limiting its ability to accurately represent bone quality and fracture risk [3–5].

Opportunistic imaging screening for bone health assessment has been investigated as an alternative to DXA, with computed tomography (CT) being the most extensively studied modality [6–8]. Opportunistic screening refers to the systematic use of imaging examinations performed for unrelated clinical indications to detect silent conditions, without additional radiation exposure or cost. This approach leverages existing imaging data to identify individuals at increased risk for low BMD and can incorporate automated analysis supported by artificial intelligence algorithms [7].

In opportunistic osteoporosis screening, lumbar spine MRI can provide indirect markers of bone marrow composition, particularly fat infiltration—a recognized hallmark of osteoporosis [3, 9]. Recent studies have proposed the vertebral bone quality (VBQ) score, derived from T1-weighted MRI, as a surrogate marker for BMD [10, 11]. The VBQ score is calculated as the ratio of the median signal intensity (SI) of the L1–L4 vertebral bodies to the SI of cerebrospinal fluid (CSF) at the L3 level. Prior research has demonstrated that VBQ can differentiate normal from osteoporotic bone with reasonable sensitivity and specificity across different clinical settings [10–17].

Most prior studies have focused on preoperative spine surgery populations [10, 14–16] or on healthy individuals across broader age ranges [11, 12, 17], leaving the performance of these approaches unexplored in older, non-surgical populations, for whom opportunistic screening may be particularly valuable. The potential of MRI to assess bone quality in other anatomical sites remains underinvestigated. Considering that the femoral neck is a critical site for osteoporotic fractures and is routinely evaluated by DXA [1, 2], exploring MRI-based metrics in this region could provide valuable complementary information.

This study aimed at determining whether signal-intensity-based metrics derived from routine lumbar spine and pelvic or hip MRI can serve as complementary opportunistic indicators of low BMD, enabling identification of individuals who may benefit from subsequent DXA referral.

## Methods

This retrospective cross-sectional observational study was in accordance with the Declaration of Helsinki and approved by the institutional ethics committee. The informed consent form was waived due to its retrospective nature. We prepared this manuscript in accordance with the Standards for Reporting Diagnostic Accuracy Studies (STARD) guidelines [18].

### Study population

We included a consecutive series of adult patients with DXA-derived *T*-scores, postmenopausal women aged ≥ 40 years and men aged ≥ 50 years, who underwent lumbar spine, hip, or pelvic MRI within 60 days of DXA between January 2019 and December 2024. Eligible participants were identified through a retrospective search of institutional records for individuals who had both DXA and MRI performed during this period. No additional sampling was necessary.

Exclusion criteria included prior surgery or fractures involving the regions of interest; focal bone lesions; hematologic or neoplastic disorders; and DXA-related artifacts such as vascular or soft tissue calcifications, spinal or hip osteoarthritis, metallic implants, retained intraluminal contrast material, and severe scoliosis or positioning errors. Patients were also excluded when femoroacetabular joint fluid was insufficient to allow reliable femoral bone quality (FBQ), when joint fluid demonstrated signal heterogeneity, or in the presence of advanced hip osteoarthritis with bone marrow edema.

### MRI protocols

All MRI examinations were performed on 1.5-T scanners (GE or Siemens) using non-contrast sequences according to institutional protocols for the lumbar spine, hip, or pelvis. Hip MRI scans were acquired with patients positioned supine, the leg extended in a neutral position, and a phased-array dedicated coil. The protocol included a coronal T1-weighted sequence (TR/TE, 761/52; thickness, 4 mm; FOV, 21 cm) and a coronal T2-weighted fat-suppressed (TR/TE, 3500/52; thickness, 4 mm; FOV, 21 cm), which were used for FBQfa and FBQcsf measurements and to confirm correct ROI placement.

Pelvis MRI examinations were performed with patients positioned supine and ankle alignment adjusted to optimize hip joint positioning. No leg support was used. Imaging was performed with a body coil. The protocol included a coronal STIR sequence (TR/TE, 4000/60; slice thickness, 5 mm; FOV, 40 cm) and a coronal T1-weighted sequence (TR/TE, 500/10; thickness, 5 mm; FOV, 40 cm) used for FBQfa and FBQcsf measurements and to confirm correct ROI placement.

Lumbar spine MRI examinations were performed with patients in the supine position, arms alongside the body, and a cushion under the legs to reduce lumbar lordosis. Imaging was acquired using either a table-integrated coil or a body coil. The protocol included a sagittal T1-weighted sequence (TR/TE, 500/10; thickness, 4 mm; FOV, 28 cm), which was used for VBQ measurements, and a sagittal T2-weighted fat-suppressed sequence (TR/TE, 3000/90; thickness, 4 mm; FOV, 28 cm), used to confirm correct CSF ROI placement.

### DXA protocol

All DXA examinations were performed on GE Lunar iDXA systems in outpatient settings, following institutional protocols in compliance with national regulatory standards. The L1–L4 vertebrae were scanned in the anteroposterior direction with participants positioned supine and the lower extremities supported to minimize lumbar lordosis. For proximal femur assessment, the hip was positioned in slight internal rotation using a dedicated positioning device to align the femoral neck parallel to the scan table. Forearm scans were performed on the non-dominant arm, with the forearm placed flat on the scanning surface and aligned with the long axis of the radius and ulna.

All participants underwent DXA of the lumbar spine and both proximal femora during the same visit, following manufacturer-recommended positioning and analysis protocols. When any of these sites was affected by artifacts or otherwise not analyzable, a non‑dominant forearm DXA was obtained to complement BMD assessment.

### Vertebral bone quality assessment

**Fig. 1:**
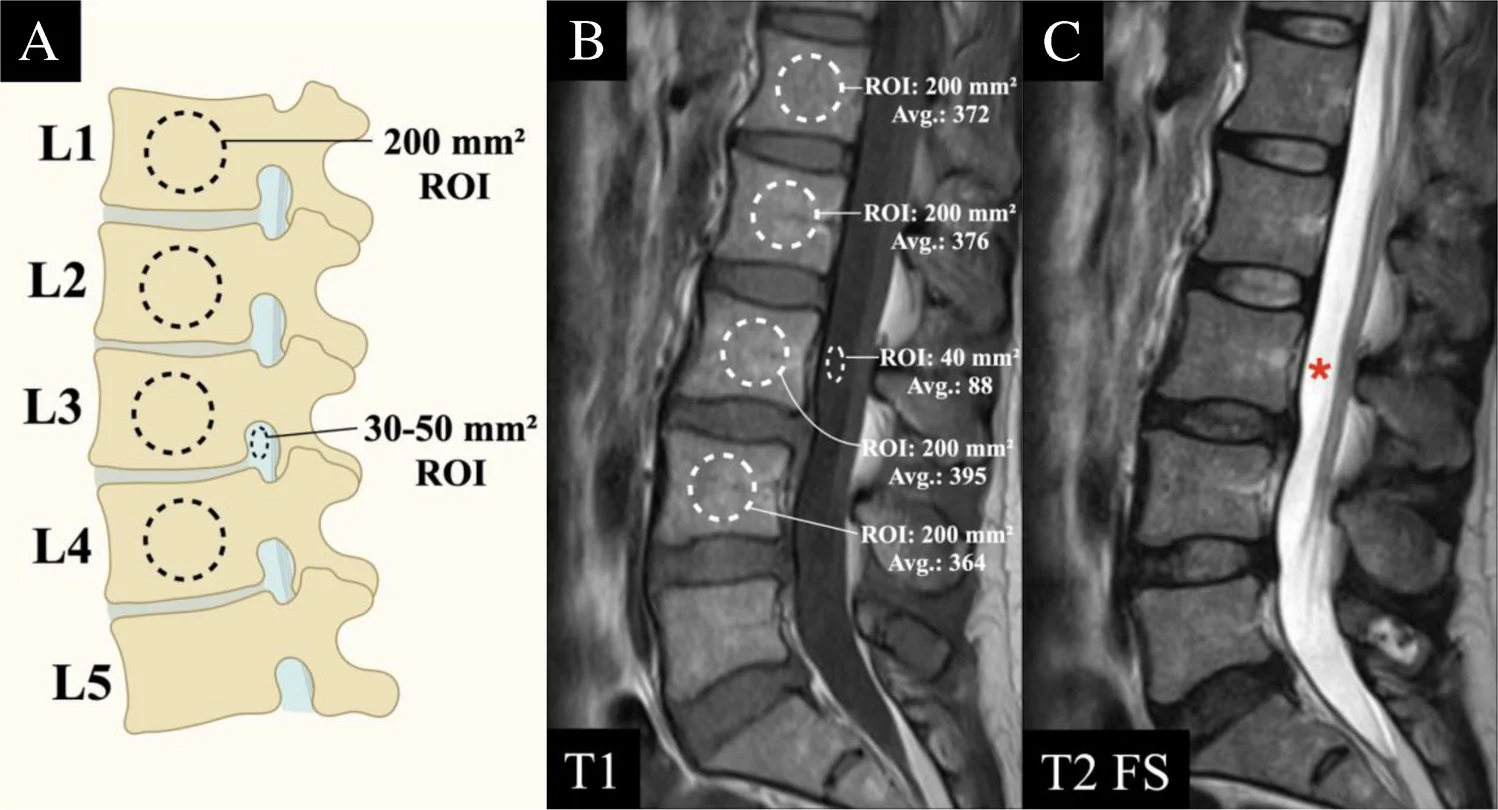
Diagram and MR images illustrating VBQ calculation. **A** Schematic illustration of the lumbar spine showing the size and placement of ROI measurements. **B** Sagittal T1-weighted lumbar spine image demonstrating ROI size, average signal intensity and placement. **C** Sagittal T2-weighted lumbar spine image; the asterisk (*) indicates the CSF ROI positioned to avoid descending nerve roots

As described by Ehresman et al. [10] and illustrated in Fig. 1, we obtained VBQ scores by placing circular regions of interest (ROIs) of 200 mm^2^ within the medullary portions of the L1–L4 vertebral bodies and a 30–50 mm^2^ ROI within the cerebrospinal fluid on midsagittal T1-weighted images. Care was taken to avoid the posterior venous plexus. The CSF ROI was positioned posterior to L3 in an area free of descending nerve roots. We calculated the VBQ score as the ratio of the median SI of the vertebral bodies to the CSF. When a vertebral body was not visible on the midsagittal cut due to scoliotic changes, a parasagittal cut was used instead.

### Femoral bone quality assessment

Building on the rationale previously applied to the lumbar spine, we propose the FBQ score to evaluate bone quality in the proximal femur. Two variations of the FBQ score were assessed for reproducibility: femoral bone quality with femoroacetabular joint fluid (FBQfa), defined as the ratio of the SI of the femoral neck to that of femoroacetabular joint fluid, and femoral bone quality with cerebrospinal fluid (FBQcsf), defined as the ratio of the SI of the femoral neck to CSF.

We obtained all FBQ measurements from coronal T1-weighted MRI acquired using hip or pelvis protocols. FBQ measurements from hip and pelvic MRI were pooled because both protocols used comparable coronal T1-weighted sequences and standardized femoral neck ROI placement. To minimize the effect of trabecular heterogeneity, FBQ was measured using a strictly standardized ROI placement protocol. Measurements were obtained on coronal T1-weighted images at the mid-femoral-neck level, defined as the slice showing the widest cancellous region. A circular 200 mm^2^ ROI was placed centrally within the medullary cavity, maintaining a ≥ 3 mm distance from the cortex and avoiding focal signal heterogeneity. ROI positioning on T1-weighted images was verified on corresponding coronal T2-weighted sequences to ensure accurate localization within homogeneous trabecular marrow. When both hips were available, bilateral measurements were performed and a single representative value per patient was selected (highest value) for statistical analysis. Examinations were excluded if the femoral neck could not accommodate the predefined ROI size. In larger femora, the ROI area remained 200 mm^2^ and was centered as described, to ensure geometric consistency and support interobserver reproducibility.

**Fig. 2:**
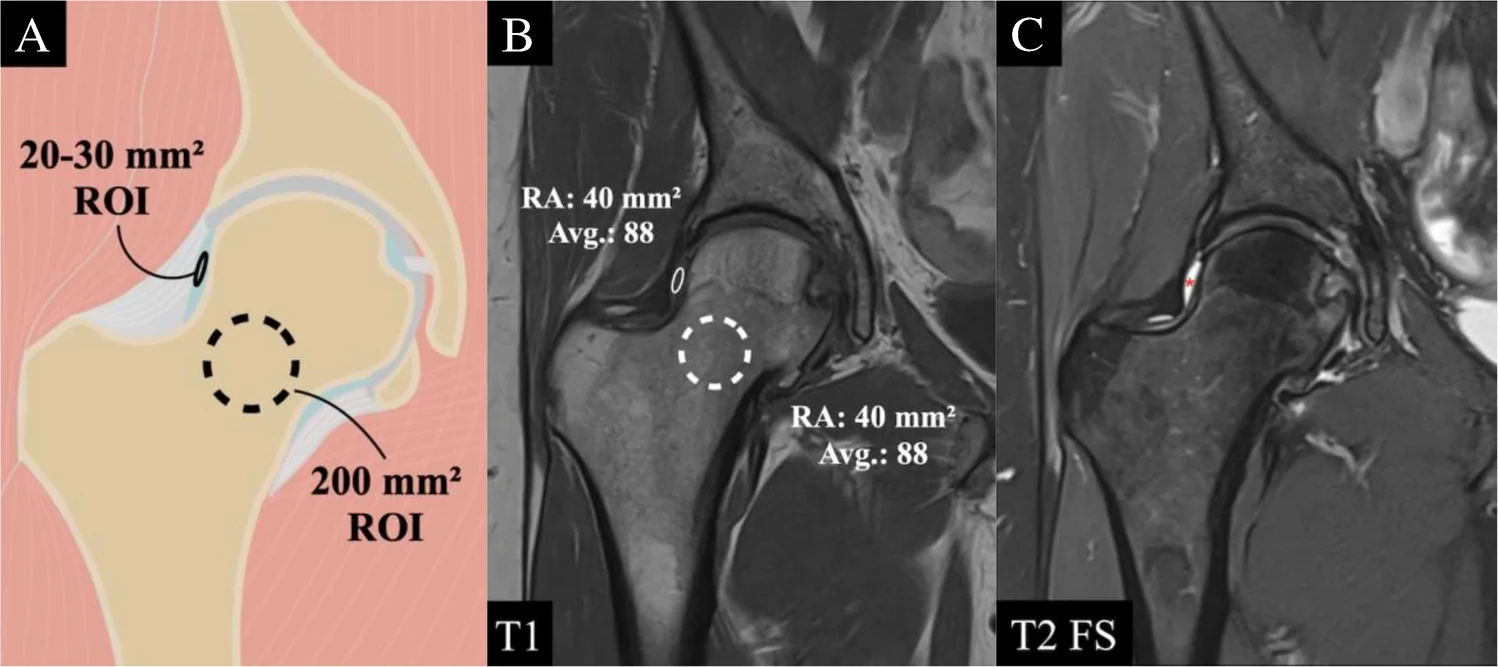
Diagram and MR images illustrating FBQfa calculation. **A** Schematic illustration of the femoroacetabular joint showing the size and placement of ROI measurements. **B** Coronal T1-weighted hip image demonstrating ROI size, average signal intensity and placement. **C** Coronal T2-weighted fat-suppressed hip image; the asterisk (*) marks the femoroacetabular joint fluid

For FBQfa, femoroacetabular joint fluid was sampled using a 20–30 mm^2^ ROI placed in the area of maximal joint effusion (Fig. 2). We performed joint fluid measurements on coronal T1-weighted images, and confirmed accurate fluid ROI placement on coronal T2-weighted sequences. Examinations with insufficient fluid, marked signal heterogeneity, or adjacent marrow abnormalities likely to affect measurement reliability were excluded. Joint fluid was selected as the reference medium because it provides a consistent, homogeneous signal on both T1- and T2-weighted sequences and is anatomically adjacent to the femoral neck. Although the bladder was considered as a potential reference, its signal intensity is highly variable due to fluctuations in urine composition, degree of filling, and possible sedimentation, making it a less reliable standard for normalization.

**Fig. 3:**
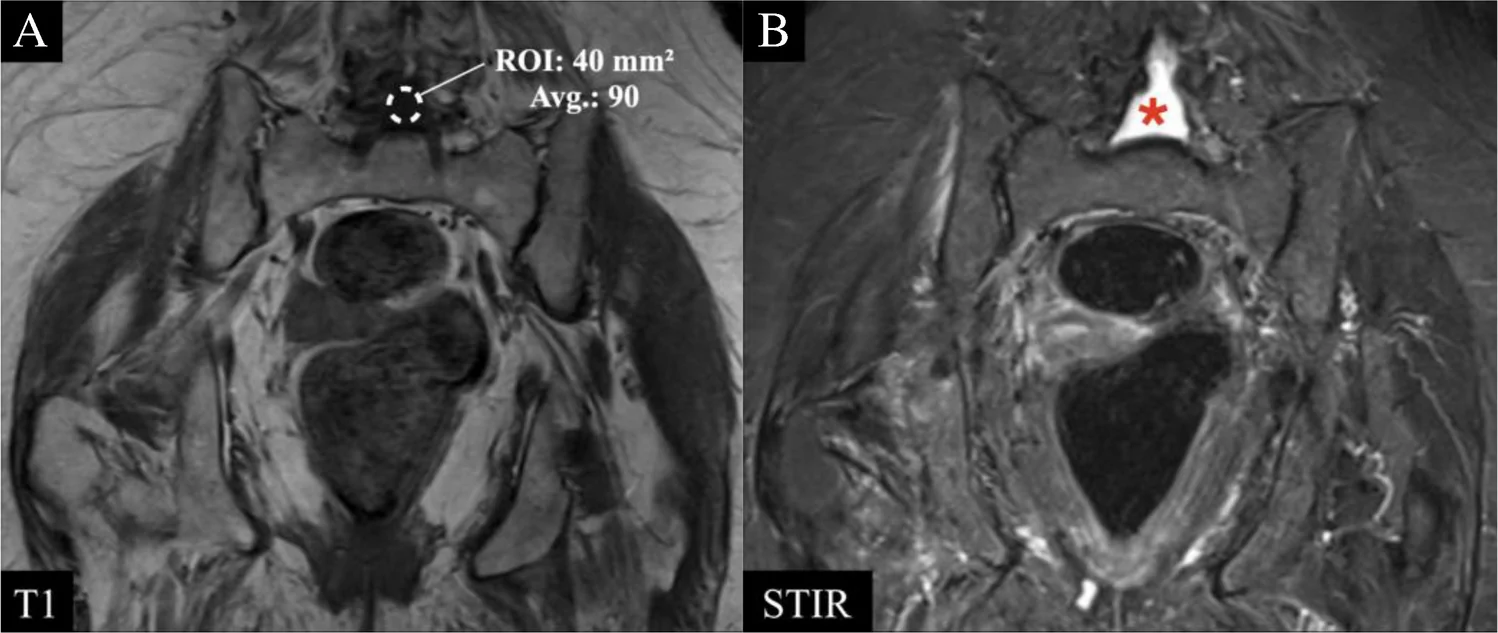
Pelvic MR images illustrating cerebrospinal fluid ROI size and placement for FBQcsf calculation. **A** Coronal T1-weighted pelvic image demonstrating ROI size, average signal intensity and placement. **B** Coronal STIR pelvic image; the asterisk (*) marks the CSF

FBQcsf could only be calculated when the imaging FOV included the lumbar spine. In these cases, an additional ROI was placed in the CSF on coronal T1-weighted images at the level of L4 or L5 (Fig. 3), standardized to an area of 30–50 mm^2^. For pelvic MRI examinations and for patients who underwent bilateral hip MRI on the same day, we obtained both FBQ measurements bilaterally, selecting the higher value for statistical analysis.

### Sample size

The sample size for VBQ and FBQ analyses was calculated to ensure 80% power and 95% confidence, assuming a correlation of 0.47 [11] and a maximum deviation of 0.10, resulting in an estimated 238 participants.

### Image analysis

We performed all measurements using the institutional picture archiving and communication system (PACS). VBQ, FBQfa and FBQcsf scores were independently assessed by two readers, blinded to DXA results and clinical data. Reader 1 was a first-year radiology resident, and Reader 2 was a musculoskeletal radiology fellow. Prior to data measurement, both readers were trained by a board-certified musculoskeletal radiologist with nine years of experience. Interobserver agreement was evaluated using a two-way random-effects ICC. DXA repeatability was not assessed because examinations were performed under standardized institutional protocols on a single-vendor platform, and duplicate scans were not available. For subsequent statistical analyses, measurements obtained by Reader 2 (the more experienced reader) were used. DXA results were obtained independently of MRI analysis. For correlation analyses, we used the lowest overall *T*-score derived from the DXA examination across all available validated skeletal sites.

### Unit of analysis

The unit of analysis was defined separately for each MRI-derived metric. For VBQ analyses, each patient contributed a single lumbar spine MRI examination. For FBQ analyses, each patient contributed a single hip or pelvic MRI examination. A subset of patients underwent both lumbar spine and hip/pelvic MRI within the inclusion period and therefore contributed to both VBQ and FBQ analyses. However, these datasets were analyzed independently, and only one observation per patient was included within each analysis. No patient contributed more than one measurement of the same type. This approach ensured independence of observations within each statistical analysis.

### Statistical analysis

Demographic data were summarized using descriptive statistics, including mean, median, and standard deviation (SD). We assessed normality of continuous variables through descriptive measures and visual inspection of histograms. Patients were classified into three groups according to World Health Organization (WHO) criteria: normal BMD (lowest *T*-score ≥  − 1), osteopenia (*T*-score between − 1.1 and − 2.4), and osteoporosis (*T*-score ≤  − 2.5). DXA was chosen as the reference standard because it is the clinically accepted gold standard for osteoporosis diagnosis according to WHO criteria [4]. Correlations with DXA were prespecified as secondary, construct-validity analyses to facilitate comparability with existing reports.

Homogeneity of variance across groups was assessed using Levene’s test. Differences in VBQ and FBQ scores among these groups were analyzed using one-way analysis of variance (ANOVA) with Bonferroni-adjusted post hoc comparisons. In addition to unadjusted analyses, Bonferroni-corrected pairwise comparisons of FBQcsf across DXA categories were repeated using a general linear model with age and sex included as covariates to account for potential confounding. Adjusted mean differences, standard errors, 95% confidence intervals, and *p* values were reported for all pairwise comparisons.

Pearson’s correlation coefficients were calculated to evaluate associations between MRI-derived bone quality scores and the lowest diagnostic DXA *T*-score. We did not perform DXA site-specific correlations. Spearman’s rank correlation coefficient was used to explore potential non-linear or monotonic relationships between FBQcsf and BMD parameters.

We assessed interobserver reliability for VBQ and FBQ measurements using a two-way random-effects ICC with 95% confidence intervals. We calculated coefficients of variation (CVs) for VBQ and FBQ to quantify relative measurement variability. The CV was defined as the standard deviation divided by the mean value and multiplied by 100, yielding a percentage measure of variability. ROC curve analysis was performed to analyze the diagnostic performance of VBQ and FBQ scores for detecting osteopenia or osteoporosis. Area under the curve (AUC) values and optimal cutoff points were derived from ROC analysis using Youden’s *J* statistic.

ROC curve analysis was performed separately for each metric according to its intended clinical target. VBQ was used to discriminate between osteoporosis from non-osteoporosis (osteopenia and normal BMD), whereas FBQcsf was used to discriminate between osteopenia and osteoporosis from normal BMD.

Subgroup analyses were performed to explore potential differences according to the MRI acquisition site. FBQcsf measurements were analyzed separately for studies acquired using pelvic MRI and hip MRI protocols. Group comparisons across DXA categories were performed using ANOVA with adjustment for age and sex. These analyses were exploratory and aimed to assess the influence of acquisition sites on FBQcsf discriminatory performance. Patients included and excluded from the FBQcsf analysis were compared using chi-square tests for categorical variables and an unpaired Student’s *t*-test for age. To evaluate whether the association between the FBQcsf and BMD differed by MRI acquisition site, a two-way ANOVA was performed including DXA category and acquisition site as main effects, as well as their interaction term.

A senior statistician with over 20 years of experience in biomedical research conducted all statistical analyses using IBM SPSS Statistics for Windows, version 22.0 (IBM Corp., Armonk, NY), and Microsoft Excel 2013. Statistical analyses were performed using a significance level (*α*) of 0.05.

## Results

**Fig. 4:**
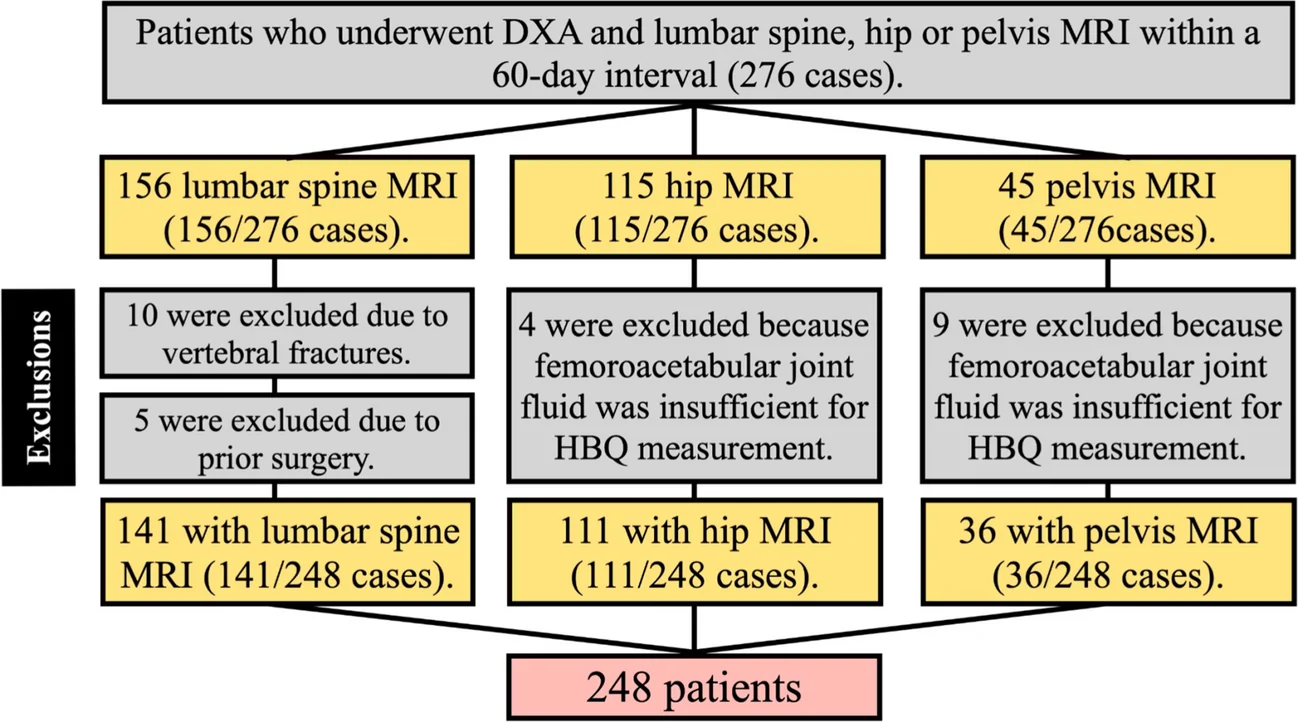
Flowchart illustrating patient selection and exclusion criteria

A total of 276 patients met the inclusion criteria, having undergone DXA and MRI of the lumbar spine, hip, or pelvis within a 60-day interval. After applying exclusion criteria, 248 patients remained eligible, comprising 288 MRI examinations: 141 lumbar spine, 111 hip, and 36 pelvis scans. We excluded 10 lumbar spine MRI due to vertebral fractures and 5 due to prior lumbar spine surgery. For hip and pelvis MRI, 13 cases were excluded because femoroacetabular joint fluid was insufficient for FBQfa measurement. Among the included patients, 21 patients underwent both lumbar spine and hip MRI, and 19 underwent lumbar spine and pelvis MRI. Based on DXA results, 79 patients (31.9%) had normal BMD, 128 (51.6%) had osteopenia, and 41 (16.5%) had osteoporosis (Fig. 4). No indeterminate DXA results were observed. Although 288 MRI examinations were available, analyses were performed separately for VBQ and FBQ metrics, each including a single observation per patient.

FBQfa was successfully obtained in all 147 hip and pelvis MRI, while VBQ was calculated in all 141 lumbar spine MRI scans. FBQcsf could be derived in only 71 cases, as its measurement required CSF to be included within the imaging FOV. Cases without CSF in the FOV were excluded from FBQcsf analysis and no imputation was performed for missing values.

### Inter‑observer agreements

VBQ score measurements demonstrated excellent reproducibility, with an ICC of 0.922 (95% CI 0.893–0.944) and a mean interobserver difference of 0.21. FBQfa measurements showed poor agreement (ICC = 0.462; 95% CI 0.340–0.567; mean difference = 0.56). When FBQcsf was calculated, reliability improved substantially (ICC = 0.874; 95% CI 0.802–0.921; mean difference = 0.44). Given the superior interobserver reliability of FBQcsf, this score was selected for statistical analysis despite the smaller sample size.

For VBQ, CV values ranged from 17.3 to 23.8% across DXA categories (mean ± SD: 3.39 ± 0.77; *n* = 141). Specifically, CVs were 20.3% in individuals with BMD, 23.8% in those with osteopenia, and 17.3% in those with osteoporosis. For FBQcsf, higher relative dispersion of measurements was observed, CV range: 23.0% to 31.0% (mean ± SD: 4.14 ± 1.18; *n* = 71). CV values were 30.6% for normal BMD, 23.0% for osteopenia, and 30.4% for osteoporosis.

### Demographic data

Patients evaluated with VBQ score had a mean age of 59.5 years (SD, 11.3; range, 40–87) and were predominantly female (87.9%). According to DXA classification, 44 patients had normal BMD, 75 had osteopenia and 22 had osteoporosis. In the FBQcsf score subgroup, there were 65 female (91.5%) and 6 male (8.5%) patients. The mean age was 61.1 years (SD, 12.2; range, 41–90). DXA classification revealed 22 patients with normal BMD, 36 with osteopenia, and 13 with osteoporosis (Table 1).

**Table 1:** Demographic characteristics and DXA classification stratified by VBQ and FBQcsf scores

	*n*	Women *n *(%)	Men *n* (%)	Age (mean ± SD)	Normal BMD *n* (%)	Osteopenia *n* (%)	Osteoporosis *n* (%)
VBQ	141	124 (87.9)	17 (12.1)	59.5 ± 11.3	44 (31.2)	75 (53.2)	22 (15.6)
FBQcsf	71	65 (91.5)	6 (8.5)	61.1 ± 12.8	22 (31.0)	36 (50.7)	13 (18.3)

Age in years

*SD* standard deviation

### Quantitative measurements

Levene’s test demonstrated no evidence of differences in variance across densitometry groups for FBQcsf (p = 0.963) and for VBQ (*p* = 0.123). For the VBQ score, mean values were 3.14 (SD = 0.64) in patients with normal BMD, 3.40 (SD = 0.81) in those with osteopenia, and 3.86 (SD = 0.67) in those with osteoporosis. The overall difference among groups was statistically significant (*p* = 0.001, Table 2). Bonferroni-adjusted analysis showed that VBQ scores were significantly higher in the osteoporosis group compared with normal BMD (mean difference = −0.72; 95% CI −1.18 to −0.25; *p* = 0.001) and compared with osteopenia (mean difference = −0.46; 95% CI −0.89 to −0.02; *p* = 0.036). There was no evidence of a difference between normal and osteopenia groups (*p* = 0.190, Table 3).

**Table 2:** Mean VBQ and FBQcsf scores across BMD categories

	BMD	Mean	SD	*N*	*p*	Effect size**
VBQ	Normal	3.14	0.64	44	0.001*	0.40
	Osteopenia	3.4	0.81	75		0.65
	Osteoporosis	3.86	0.67	22		0.45
FBQcsf	Normal	3.54	1.09	22	0.002*	1.18
	Osteopenia	4.61	1.06	36		1.13
	Osteoporosis	3.84	1.17	13		1.36

*SD* standard deviation

*ANOVA test

**Effect size expressed as Cohen’s *d*

**Table 3:** Comparisons of VBQ and FBQcsf scores across BMD categories

	Comparison	Mean Difference	Standard Error	*p*	95% CI	Effect size**
VBQ	Normal and osteopenia	−0.26	0.14	0.190*	−0.60 to 0.08	0.347
	Normal and osteoporosis	−0.72	0.19	0.001*	−1.18 to −0.25	1.091
	Osteopenia and osteoporosis	−0.46	0.18	0.036*	−0.89 to −0.02	0.579
FBQcsf	Normal and osteopenia	−1.06	0.29	0.002*	−1.79 to 0.34	0.977
	Normal and osteoporosis	−0.29	0.38	> 0.999*	−1.23 to 0.64	0.256
	Osteopenia and osteoporosis	0.77	0.35	0.096*	−0.09 to 1.63	0.693

*Bonferroni-adjusted pairwise comparisons

**Effect size expressed as Cohen’s *d*

For the FBQcsf score, mean values were 3.54 (SD = 1.09) in the normal group, 4.61 (SD = 1.06) in the osteopenia group, and 3.84 (SD = 1.17) in the osteoporosis group, with a significant overall difference among all groups (*p* = 0.002, Table 2). In unadjusted Bonferroni-corrected pairwise comparisons, FBQcsf values were significantly higher in individuals with osteopenia compared with those with normal BMD (mean difference =  − 1.06; *p* = 0.002). There was no evidence of difference between the normal BMD and osteoporosis groups (*p* > 0.999) or between the osteopenia and osteoporosis groups (*p* = 0.096; Table 3). After repeating the Bonferroni-adjusted multiple comparison analysis with adjustment for age and sex, FBQcsf remained significantly higher in the osteopenia group compared with normal BMD (mean difference =  − 1.94; *p* = 0.001). The comparison between osteopenia and osteoporosis also reached statistical significance, with higher FBQcsf values observed in osteopenia (mean difference = 1.32; *p* = 0.011), and there was no evidence of a difference between normal BMD and osteoporosis (*p* = 0.606).

FBQcsf demonstrated a statistically significant negative correlation with the lowest BMD measured by DXA (*r* =  − 0.274, *p* = 0.021). FBQcsf showed an inverse trend with the lowest DXA *T*-score, but there was no evidence of an association (*r* =  − 0.139, *p* = 0.248).

### Diagnostic performance

**Fig. 5:**
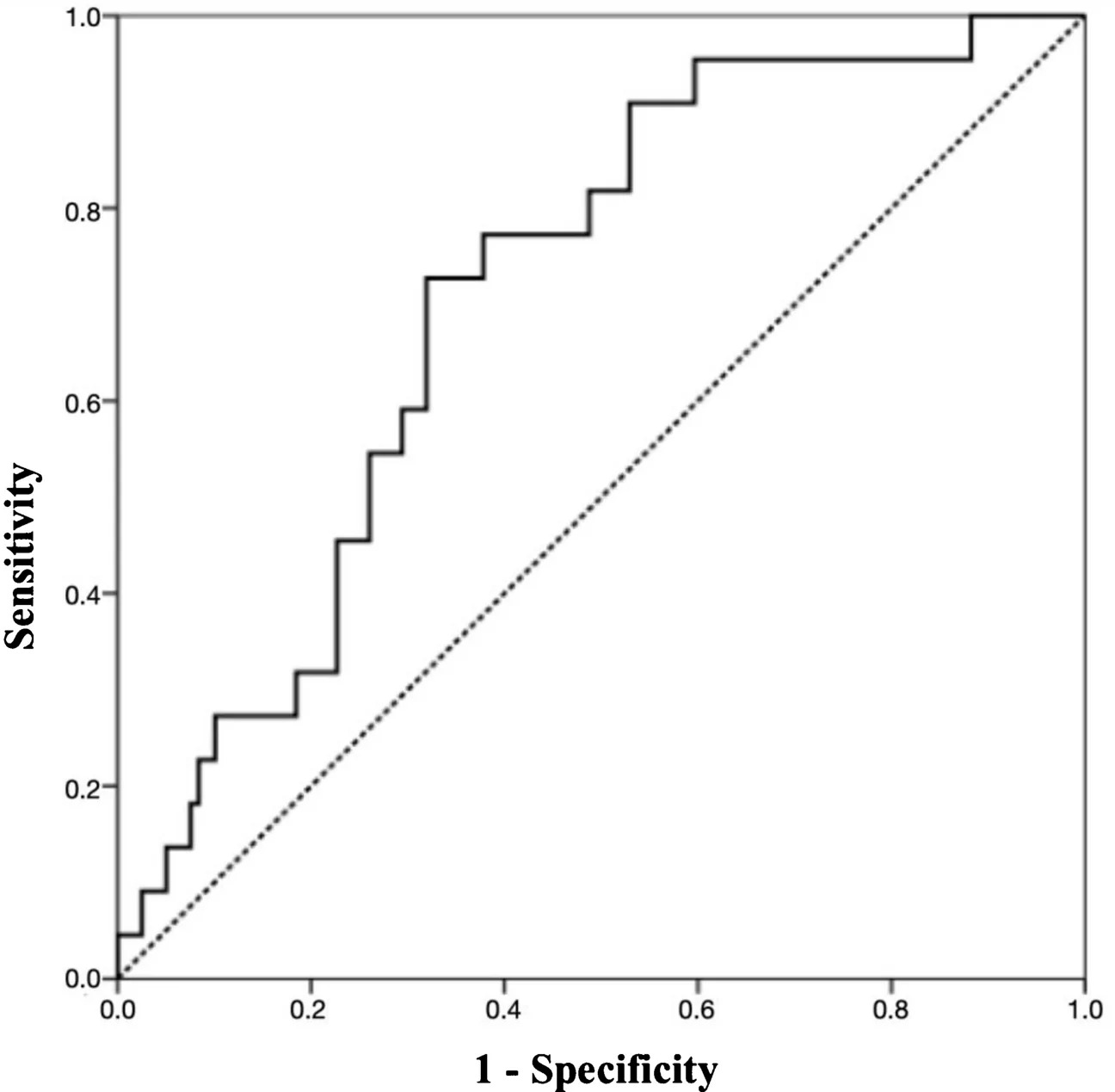
ROC curve showing the discriminatory ability of the VBQ (black line) predicting osteoporosis

We performed ROC analysis to evaluate the ability of VBQ and FBQcsf scores to discriminate between bone mineral density categories. For VBQ, the area under the curve for identifying osteoporosis from osteopenia and normal BMD was 0.71 (95% CI 0.61–0.81), indicating moderate discriminatory performance (Fig. 5). The optimal cutoff value determined by the Youden index was 3.61, yielding a sensitivity of 72.7% and specificity of 68.1% (Table 4).

**Table 4:** Discriminatory performance of VBQ and FBQcsf scores for identifying osteoporosis and osteopenia, respectively

Score	AUC (95% CI)	Cutoff	Sensitivity % (95% CI)	Specificity % (95% CI)
VBQ	0.71 (0.61–0.81)	3.61*	72.7 (49.8–89.3)	68.1 (58.9–76.3)
FBQcsf	0.71 (0.58–0.84)	3.33*	85.7 (72.8–94.1)	50 (28.2–71.8)

*Youden’s index

**Fig. 6:**
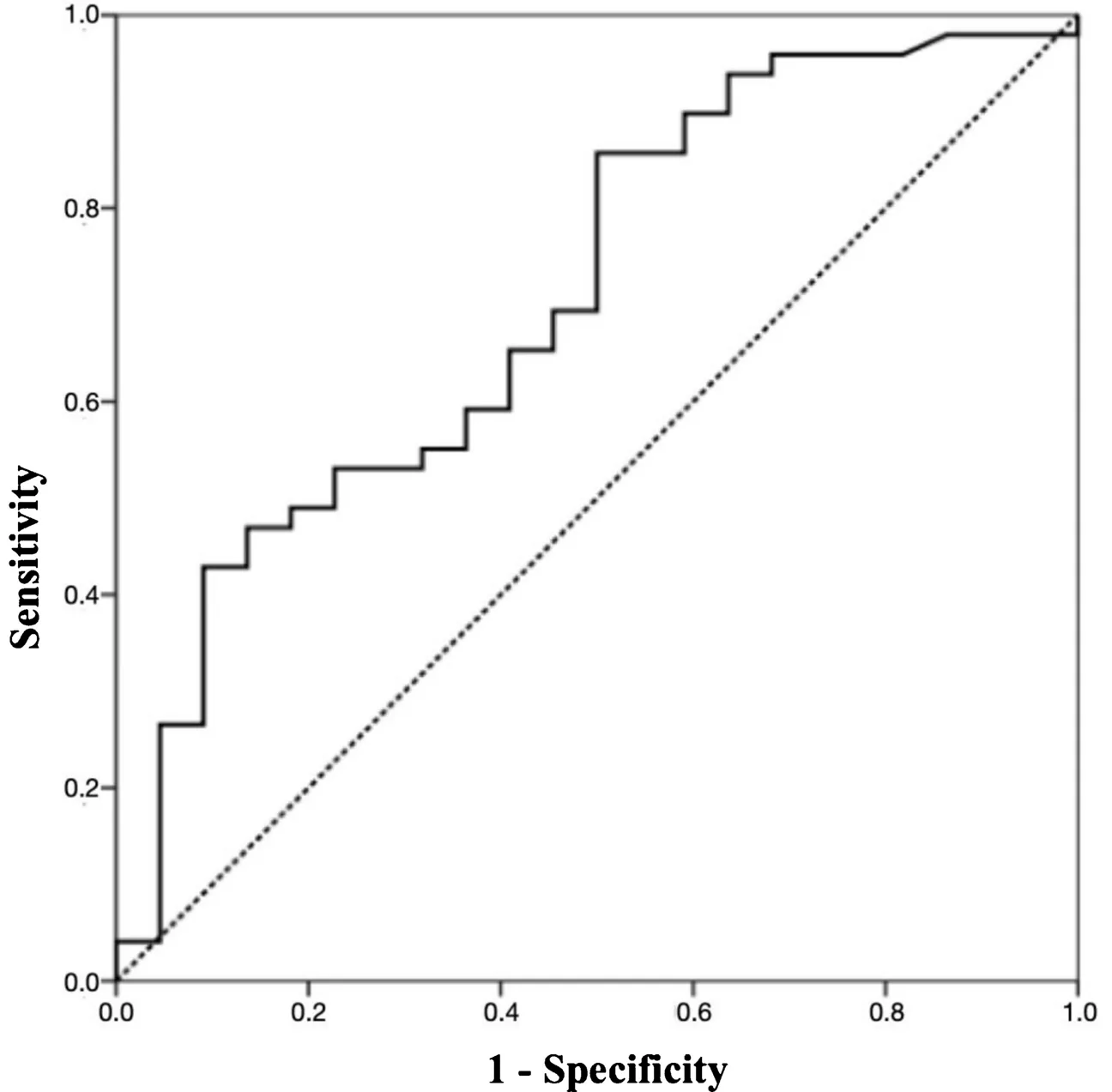
ROC curve illustrating the discriminatory performance of the FBQcsf score (black line) for distinguishing patients with osteopenia from those with normal BMD

For FBQcsf, the AUC for distinguishing patients with osteopenia from those with normal BMD was 0.71 (95% CI 0.58–0.84) (Fig. 6). The best cutoff value was 3.33, with a sensitivity of 85.7% and specificity of 50.0% (Table 4).

### Correlation analysis

**Fig. 7:**
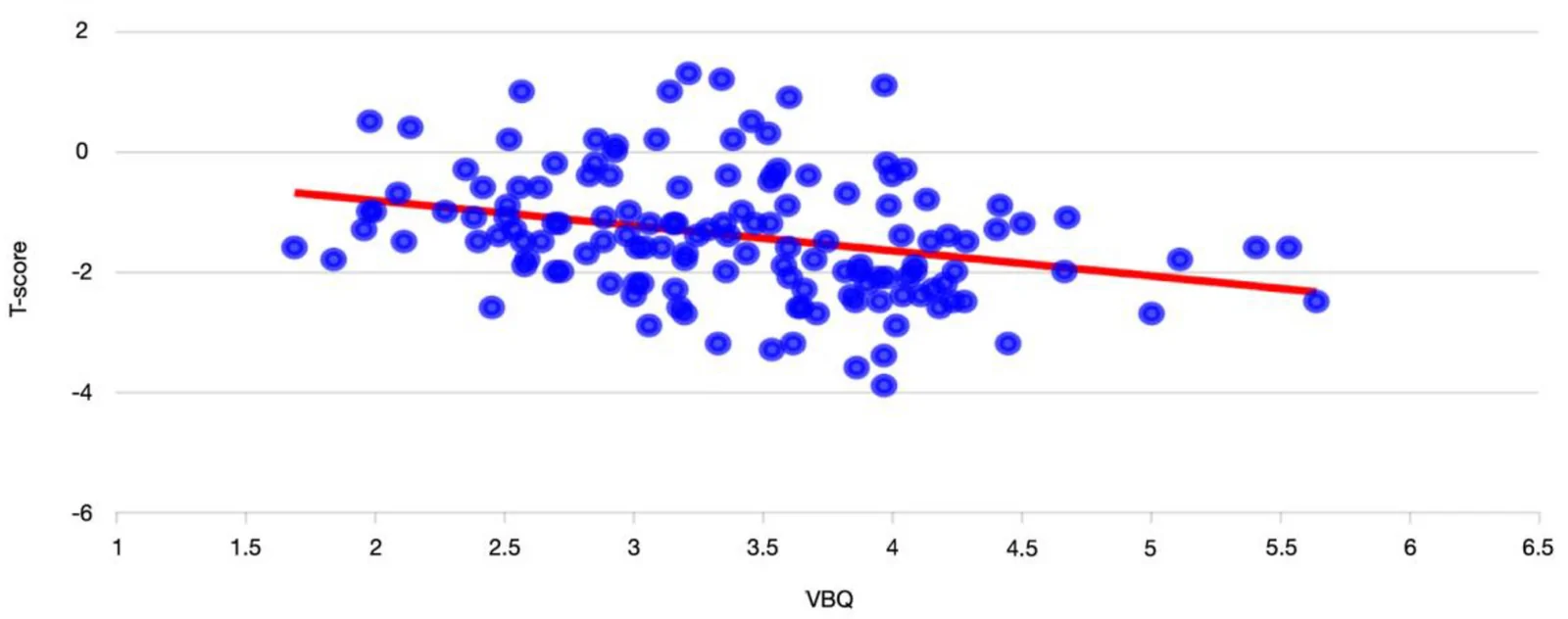
Scatter plot showing correlation between VBQ score and DXA *T*-score

Pearson correlation analysis demonstrated a weak-to-moderate negative association between VBQ score and DXA lowest *T*-score (*r* = –0.307; *p* = 0.002; *n* = 141), indicating that higher VBQ values are associated with lower BMD (Fig. 7). In contrast, FBQcsf showed no evidence of correlation with the *T*-score (*r* = –0.172; *p* = 0.151; *n* = 71; supplementary Table 5). Non-parametric analysis using Spearman correlation yielded comparable results (supplementary Table 5), supporting the robustness of the observed associations. Discriminatory performance tables for VBQ and FBQcsf cut-offs are provided in supplementary Table 6 and 7.

When FBQcsf measurements were analyzed separately according to the MRI acquisition site, heterogeneous patterns were observed, without consistent statistical significance across acquisition sites. In the pelvic MRI subgroup, FBQcsf values demonstrated a statistically significant overall difference across DXA categories after adjustment for age and sex (*p* = 0.013), with higher values observed in individuals with osteopenia. FBQcsf values derived from hip MRI did not provide evidence of an overall group difference after adjustment (*p* = 0.145; supplementary Table 8). Two-way ANOVA demonstrated no significant interaction between DXA category and acquisition site (*p* = 0.498), indicating that the relationship between the FBQcsf and bone density status did not differ across hip and pelvic subgroups.

Comparison between patients included and excluded from the FBQcsf analysis showed no significant differences in age, sex distribution, or DXA categories. As expected, MRI acquisition site differed significantly (*p* < 0.001), with all excluded patients undergoing hip MRI, whereas included patients had hip or pelvic examinations with CSF included in the field of view (supplementary Table 9).

## Discussion

In this retrospective study, we evaluated MRI-derived SI ratios as opportunistic markers of BMD in patients at risk for osteoporosis. The VBQ score demonstrated excellent interobserver reliability and a weak-to-moderate inverse correlation with DXA *T*-scores, with moderate discriminatory performance for identifying osteoporosis. The FBQcsf demonstrated high reproducibility and was able to distinguish individuals with osteopenia from those with normal BMD, a distinction not observed with VBQ. Although its correlation with DXA* T*-scores was non-significant, the higher sensitivity of FBQcsf for osteopenia suggests potential utility for detecting early bone quality changes not fully reflected by DXA.

We included postmenopausal women over 40 years and men over 50 years without a history of lumbar spine surgery, while prior studies primarily examined healthy individuals aged over 18 years [11, 12, 17] or surgical lumbar spine cohorts [10, 14–16]. Targeting a population with a higher likelihood of osteoporosis increases event rates and enhances statistical power for correlation analyses, enabling a more robust evaluation of VBQ as an opportunistic screening marker.

In our analysis, VBQ demonstrated excellent interobserver agreement (ICC = 0.922) and a weak-to-moderate negative correlation with DXA *T*-scores (*r* = −0.31). Previous studies in surgical cohorts have consistently shown that VBQ can distinguish normal from osteoporotic bone, with stronger correlations to DXA or QCT-derived *T*-scores (*r* ranging from −0.358 to −0.510) [10, 14–16]. In contrast, Özmen et al. evaluated VBQ in healthy individuals over 18 years and reported weak-to-moderate negative correlations with DXA *T*-scores (*r* = −0.26) [17]. Our results are consistent with those reported by Özmen et al., as the correlation observed in our older, non-surgical cohort was modest, lower than values reported in surgical populations [11, 17].

The CV values reported for MRI-derived metrics reflect the relative variability of measurements across individuals within each DXA category. Accordingly, the higher CVs observed for FBQcsf likely reflect greater biological and measurement variability within this subgroup.

Our ROC analysis indicated moderate discriminatory performance for osteoporosis detection (AUC = 0.71), with a cutoff of 3.61 yielding balanced sensitivity (72.7%) and specificity (68.1%). These results are consistent with previous reports showing AUC values between 0.67 and 0.81 and cutoff thresholds ranging from 2.6 to 3.6, reinforcing the robustness of VBQ across diverse cohorts [10–12, 14–17]. Despite variability in correlation strength, VBQ has shown excellent reproducibility and discriminatory performance, supporting its potential role as an opportunistic screening marker for bone quality assessment. Importantly, the optimal cutoffs were derived and evaluated within the same dataset, which may lead to optimistic estimates of discriminatory performance. External validation in independent cohorts is necessary to confirm the generalizability of these thresholds.

The ROC analyses of VBQ and FBQcsf were performed for different clinical targets. VBQ was used to identify osteoporosis versus non-osteoporosis, while FBQcsf was used to distinguish osteopenia and osteoporosis versus normal BMD. These represent distinct diagnostic tasks along the spectrum of bone loss and the corresponding AUC values should not be directly compared as equivalent measures of performance.

Although VBQ values showed a progressive increase from normal BMD to osteopenia and osteoporosis with statistically significant differences, Bonferroni post hoc testing did not distinguish normal BMD from osteopenia, limiting its clinical discriminatory power, consistent with previous reports [11, 12, 14, 15].

Femoral bone quality scores exhibited variable performance. Among the two methods assessed, FBQcsf demonstrated superior reproducibility (ICC = 0.874) compared with FBQfa (ICC = 0.462). Due to its higher interobserver reliability, FBQcsf was selected for statistical analysis despite the smaller sample size. The poor agreement observed with FBQfa likely reflects variability in joint effusion volume and the inherent difficulty of obtaining consistent ROIs in small fluid collections. When CSF served as the reference signal (FBQcsf), reliability improved substantially, consistent with prior VBQ literature that emphasizes the stability and homogeneity of CSF signal on T1-weighted imaging [10, 14–16].

FBQcsf demonstrated a significant association with DXA-derived BMD, while its correlation with the lowest DXA *T*-scores did not reach statistical significance. The lack of statistical significance for the FBQcsf–*T*-score correlation may reflect limited statistical power. The sample size, particularly within individual diagnostic categories, may have been insufficient to detect modest associations after categorization, raising the possibility of a type II error. This interpretation is supported by the fact that the correlation coefficients for BMD and *T*-score were directionally concordant, suggesting a consistent biological relationship that did not fully manifest at the categorical level.

We observed higher FBQcsf values in individuals with osteopenia than in those with osteoporosis. While the overall unadjusted group comparison was significant, pairwise differences between osteopenia and osteoporosis did not reach statistical significance, likely reflecting sample size limitations and variability within the osteoporosis subgroup. After adjustment for age and sex, FBQcsf values remained significantly higher in osteopenia compared with normal BMD, and importantly, the difference between osteopenia and osteoporosis became statistically significant. This suggests that demographic factors partially obscured group-level differences in the unadjusted analysis. These findings suggested a potential non-linear relationship between FBQcsf and the densitometry categories. Additionally, no significant association was observed on either Spearman’s or Pearson’s analyses between FBQcsf and the lowest DXA *T*-score, indicating the absence of a linear or monotonic relationship. Together, these results suggest that, unlike VBQ, FBQcsf may exhibit a non-linear and non-monotonic relationship with DXA-derived categories.

Unlike VBQ, FBQcsf distinguished individuals with normal BMD from those with osteopenia. This difference may reflect physiological variations in marrow composition between the femur and the vertebrae. Hematopoietic marrow is progressively replaced by fatty marrow beginning in long bones such as the femur, while vertebrae retain hematopoietic activity for longer periods [19]. In adults with osteopenia or osteoporosis, marrow adiposity increases at both sites, but imaging and MR spectroscopy studies confirm that fat accumulation occurs earlier and more extensively in the proximal femur, particularly in older individuals and those with low BMD [20, 21]. Early fatty transformation is associated with reduced BMD, microstructural deterioration, and increased risk of hip fractures, positioning marrow adiposity as a potential early marker of skeletal fragility [20, 22, 23].

Our FBQcsf findings align with those of Shen et al., who reported an inverse relationship between pelvic bone marrow adipose tissue measured by MRI and BMD assessed by DXA in both younger and older adults. These observations indicate that increased marrow adiposity is associated with compromised bone integrity, likely reflecting a shift in mesenchymal stem cell differentiation toward adipogenesis rather than osteoblastogenesis. While Shen et al. provided volumetric evidence of this association, FBQcsf offers a practical, signal-based metric that can be derived opportunistically from routine hip MRI, reinforcing the clinical relevance of marrow fat as a determinant of skeletal fragility [24].

Collectively, these findings raise the hypothesis that FBQcsf may be particularly sensitive to early marrow compositional changes characteristic of osteopenia. In advanced osteoporosis, additional microarchitectural deterioration and increased marrow heterogeneity may alter signal distributions within the region of interest, potentially attenuating the discriminatory performance of signal-based metrics. Within this framework, femoral MRI-derived bone quality assessment may complement spine-based approaches and DXA measurements, particularly in the context of opportunistic screening aimed at identifying early skeletal alterations in at-risk populations.

Subgroup analyses stratified by MRI acquisition site suggested that FBQcsf performance may vary between pelvic and hip MRI examinations. FBQcsf derived from pelvic MRI demonstrated statistically significant group-level differences, whereas measurements derived from hip MRI did not. These findings should be interpreted cautiously given the limited sample size and potential heterogeneity between subgroups, including variations in patient characteristics, imaging protocols and field‑of‑view coverage. Although exploratory, these findings highlight the importance of acquisition-specific considerations when interpreting MRI-based bone quality metrics.

In our study, the absence of a significant correlation between FBQcsf and the lowest DXA *T*-score, together with its ability to discriminate normal BMD from osteopenia, suggests that the relationship between FBQcsf and DXA may not follow a consistent monotonic pattern across disease stages. Accordingly, FBQcsf may be best interpreted as a stage-sensitive marker reflecting marrow-related alterations rather than a linear surrogate of BMD. Further studies incorporating longitudinal designs, fracture outcomes, and analytical approaches tailored to non‑linear associations are warranted.

Opportunistic imaging has emerged as a promising strategy to address the diagnosis of osteoporosis. Most initiatives in the literature have focused on CT, where opportunistic screening using attenuation values or automated algorithms has demonstrated strong correlation with BMD and fracture risk [6–8]. Our study exemplifies the application of this concept to MRI, a modality widely used in older adults. Unlike CT, MRI offers the advantage of avoiding ionizing radiation and is frequently performed for spine and pelvic evaluations, providing an ideal opportunity for secondary analysis without additional cost or patient burden [9, 14]. This research expands the scope of opportunistic screening beyond CT and demonstrates the feasibility of integrating bone health assessment into routine MRI interpretation. This approach enhances accessibility and aligns with principles of value-based care, potentially enabling earlier detection and intervention in at-risk individuals.

This study has several strengths. First, it introduces a novel femoral bone quality score (FBQcsf) alongside the established VBQ, extending MRI-based bone quality assessment beyond the spine to the proximal femur, a critical site for osteoporotic fractures and a standard DXA evaluation region. Second, the analysis focuses on an older, non-surgical population, the group most likely to benefit from opportunistic osteoporosis screening, thereby enhancing clinical applicability. Third, the study demonstrates excellent interobserver reproducibility for VBQ (ICC = 0.922) and FBQcsf (ICC = 0.874), confirming the robustness of these MRI-derived metrics. Fourth, by leveraging routine MRI without additional radiation or cost, it highlights the feasibility of integrating opportunistic screening into standard workflows. Finally, adherence to STARD guidelines ensures methodological rigor and transparency.

This study also has several limitations. FBQcsf could only be measured in a subset of examinations where CSF was included in the FOV, reducing sample size and statistical power. Our cohort was predominantly female; therefore, estimates in men are less precise and sex-specific generalizability is limited. This sex imbalance reflects typical referral patterns and the higher prevalence of osteoporosis among postmenopausal women, but it constrains external validity. In addition, the dataset was not perfectly balanced, with relatively fewer patients classified as osteoporotic by DXA. Although this distribution mirrors real-world populations undergoing routine MRI, the limited number of osteoporotic cases likely reduced statistical power for subgroup analyses and resulted in wider confidence intervals for discriminatory performance estimates. Nevertheless, homogeneity of variance across groups was confirmed, including in the FBQcsf subset, and results remained consistent in nonparametric analyses, supporting the robustness of the findings.

Although MRI examinations were performed across different anatomical regions, signal-based metrics are sensitive to protocol and manufacturer variability, and standardization across scanners was not formally assessed. Demographic and clinical factors that could influence VBQ and FBQ measurements, such as body mass index, steroid use, alcohol consumption, hyperlipidemia, and diabetes were not evaluated. FBQcsf measurements were obtained from both pelvic and hip MRI protocols. While exploratory subgroup analyses were performed, limited sample sizes reduce statistical power and preclude definitive conclusions regarding acquisition-specific performance. Femoral positioning was protocol-dependent and not rigidly standardized across hip and pelvic MRI examinations. This may introduce minor variability in proximal femoral rotation and, consequently, in femoral-neck ROI orientation on MRI. Although we sought to mitigate this effect through standardized central medullary ROI placement, cross-sequence verification, and bilateral sampling when available, residual positioning-related variability cannot be fully excluded. Larger, prospectively balanced cohorts are needed to clarify the impact of MRI protocol and acquisition site on FBQcsf behavior.

Both VBQ and FBQ measurements are inherently influenced by trabecular and marrow heterogeneity, as small ROIs may disproportionately sample focal microstructural patterns, partial-volume interfaces, or localized variations in marrow signal. Although ROI size and placement were predefined, this cannot completely eliminate sampling variability. We did not morphologically assess hematopoietic marrow content or its spatial heterogeneity within the proximal femur. This unmeasured heterogeneity may have attenuated linear associations of FBQcsf with DXA and contributed to wider confidence intervals. Future investigations should explore voxel-wise or multi-site averaging approaches, incorporate quantitative marrow composition techniques and assess external reproducibility across scanners and readers to further improve robustness.

The study was not designed to evaluate prevalent or incident fractures; therefore, comparisons with DXA should be interpreted as contextual instead of definitive validation. Fracture-based outcomes and dedicated bone quality assessments will be required to establish the clinical utility of VBQ and FBQ and represent important directions for future research.

In summary, VBQ demonstrated excellent reproducibility and moderate discriminatory performance for detecting osteoporosis in our predominantly postmenopausal female cohort. However, variability in cutoff values across cohorts reinforces the need for prospective multicenter studies and automated measurement tools to validate these findings and establish standardized thresholds [10, 11, 14–17]. FBQcsf showed significant association with DXA-derived BMD and moderate discriminatory performance for differentiating patients with osteopenia from those with normal BMD.

Given the absence of fracture outcomes, the use of a selected FBQcsf subset, and the lack of external validation of ROC-derived thresholds, VBQ and FBQcsf should be considered exploratory opportunistic imaging biomarkers rather than diagnostic tools. In this stage, they may help identify individuals at risk for low BMD, who could benefit from formal DXA assessment, but their clinical utility, optimal thresholds, and generalizability require validation in prospective multicenter studies incorporating fracture outcomes. Future research should focus on evaluating these metrics in diverse populations, defining thresholds, and assessing predictive value for clinical outcomes such as fracture risk. If validated, such approaches may contribute to the development of opportunistic, workflow-integrated screening strategies, although their impact on clinical practice and healthcare systems remains to be determined.

## Supplementary information

Below is the link to the electronic supplementary material.

ESM 1 (DOCX 19.2 KB)

## Data Availability

Data are available upon reasonable request.
