# Supplementary material for: Routine MRI for opportunistic osteoporosis screening: diagnostic accuracy of vertebral bone quality and a novel femoral bone quality score compared with DXA
Source: Skeletal Radiol. 2026 Aug 8;55(11):2953–65. doi: 10.1007/s00256-026-05330-z (PMC13574861; doi:10.1007/s00256-026-05330-z)
Supplement: Supplementary file 1 [file 256_2026_5330_MOESM1_ESM.docx]

Supplementary material

Supplementary table 5. Pearson and Spearman Correlations Between VBQ and FBQcsf with the lowest DXA T-Score.

| Score | N | r | p | ρ | p |
| --- | --- | --- | --- | --- | --- |
| VBQ | 141 | -0.307 | <0.001 | -0.35 | <0.001 |
| FBQcsf | 71 | -0.172 | 0.151 | -0.139 | 0.248 |

r: Pearson Correlation Coefficient; ρ: Spearman rank correlation coefficient

Supplementary table 6. Discriminatory performance of VBQ cut-off value for osteoporosis screening

|  | Osteoporosis | Normal BMD |
| --- | --- | --- |
| VBQ ≥ 3.61 | 16 (true positive) | 38 (false positive) |
| VBQ < 3.61 | 6 (false negative) | 81 (true negative) |

Supplementary table 7. Discriminatory performance of FBQcsf cut-off value for osteopenia screening

|  | Osteopenia | Normal BMD |
| --- | --- | --- |
| FBQcsf ≥ 3.33 | 31 (true positive) | 11 (false positive) |
| FBQcsf < 3.33 | 5 (false negative) | 11 (true negative) |

Supplementary table 8. FBQcsf values across DXA categories according to MRI acquisition site

|  | DXA | Mean | N | p | p |
| --- | --- | --- | --- | --- | --- |
| FBQcsf (hip and pelvis) | Normal BMD  Osteopenia  Osteoporosis  Total | 3.54  4.61  3.84  4.14 | 22  33  13  71 | 0.002* | <0.001** |
| FBQcsf (pelvis) | Normal BMD  Osteopenia  Osteoporosis  Total | 3.83  5.13  4.25  4.62 | 10  20  13  36 | 0.013* | 0.003** |
| FBQcsf (hip) | Normal BMD  Osteopenia  Osteoporosis  Total | 3.30  3.96  3.48  3.64 | 12  16  7  35 | 0.145* | 0.213** |

*ANOVA test; **Linear model adjusted for age and sex

Supplementary table 9. Comparison between patients included and excluded from the FBQcsf

| Variable | Included (n = 71) | Excluded (n = 76) | p |
| --- | --- | --- | --- |
| Age, mean ± SD | 60.8 ± 12.2 | 62.4 ± 12.3 | 0.411* |
| Sex  Female, n (%)  Male, n (%)    DXA category  Normal, n (%)  Osteopenia, n (%)  Osteoporosis, n (%)  MRI  Pelvis, n (%)  Hip, n (%) | 65 (91.5)  6 (8.5)      22 (31)  36 (50.7)  13 (18.3)  36 (50.7)  35 (49.3) | 69 (90.8)  7 (9.2)      22 (28.9)  42 (55.3)  12 (15.8)  0 (0)  76 (100) | 0.871**  0.847**  < 0.001** |

*SD* standard deviation; Age in years; *Unpaired Student's t-test; **Chi-square test
